# Mobile perceived trust mediation on the intention and adoption of FinTech innovations using mobile technology: A systematic literature review

**DOI:** 10.12688/f1000research.74656.2

**Published:** 2022-03-02

**Authors:** Hatim M. Dawood, Chee Yoong Liew, Teck Chai Lau

**Affiliations:** 1 UCSI Graduate Business School, Faculty of Business & Management, UCSI University, Kuala Lumpur, KL, Malaysia

**Keywords:** Mobile Perceived Trust, Perceived Risk, Fintech, Perceived Benefit, Net Valence framework, Mobile technology acceptance model, Benefit-Risk framework.

## Abstract

The banking and financial sectors have witnessed a significant development recently due to financial technology (FinTech), and it has become an essential part of the financial system. Many factors helped the development of this sector, including the pandemics such as Covid-19, the considerable increasing market value of the FinTech sector worldwide, and new technologies such as blockchain, artificial intelligence, big data, cloud computing and mobile technology. Moreover, changes in consumer's preferences, especially the Z-generation (digital generation). FinTech shifted the traditional business models to mobile platforms characterized by ease of access and swift transactions. Mobile technology became the main backbone for FinTech innovations and acts as a channel to deliver FinTech services that overcome all geographical and timing barriers, thus enhancing financial inclusion. Mobile perceived Trust (MPT), or the trust in using financial business models via mobile technology, is a crucial factor in the FinTech context that has mediation effects on the intention and adoption of different FinTech business models. Unfortunately, few studies have explored MPT mediations on consumers' intention to adopt FinTech innovations using mobile technology. Typically, many studies examined trust/MPT as an independent and unidirectional variable and investigated its effects on behaviour intention without predicting its mediation effects. This study aimed to develop a systematic literature review on MPT mediation in FinTech, focusing on the period from 2016 and 2021, in journals ranked Q1 and Q2, and known-based theories such as the technology acceptance model, the unified theory of acceptance and use of technology, and the mobile technology acceptance model. This study found that only four articles were published in Q1 and Q2 journals. In these articles, the MPT was used as a mediator, and its effects were measured on the intention and adoption of the behaviour.

## Introduction

One of the consequences of financial technology (FinTech) platforms using mobile technology is that their risk affects consumer trust and prevent adopting this type of technology-driven business model worldwide. Peer to peer (PTP) lending, crowdfunding and invoice funding are examples of FinTech credit facilitated by electronic platforms ( Lenz, 2016) to offer complete lending transactions ( Yuwei, Zhihan, & Bin, 2017) and allow consumers to perform credit transactions ( Lenz, 2016; Suryono, Purwandaria, & Budia, 2019). The credit transactions on the PTP lending platform includes buying loans from the lenders or creditors such as financial institutions ^1^ ( Lenz, 2016). Buying loans from PTP platforms reduces the borrower's credit obligations imposed by traditional financial institutions, financial regulators, and authorities. Thus, the restrictions on loans are reduced, and it is easier for the borrower to obtain them in a shorter time than usual and within non-strict, flexible credit restrictions, disrupting the traditional financial value chain ( Ryu, 2018; Ryu & Ko, 2020).

Scholars introduced perceived risk as to the magnitude of uncertainty on the results of innovation usage ( Tan & Leby, 2016; Farah, 2017; Ryu & Ko, 2020). Scholars have identified many risks in the financial technology field, such as financial, legal, security, and operational risks ( Ryu & Ko, 2020), which act as barriers for financial institutions. For example, PTP lending platforms welcome borrowers (debtors), such as individuals and small and medium-sized enterprises, who may be categorized as high credit risk and have already been rejected by banks and other finance companies due to the differences in credit risk assessment ( Lenz, 2016; Ozili, 2018). These innovations contain advanced technologies that change the nature of the operation and behaviour of the usual business models.

Perceived trust reflects a person's belief that the use of m-commerce and similar technologies are secure and have privacy threats ( Tang, Zhang, & Akram, 2019). Moorman et al. (1993) defined trust as a willingness to depend on a partner in whom one has self-confidence. Lack of trust has confirmed that trust is the most significant long-term barrier to a financial system’s success ( Gao & Waechter, 2015). Perceived trust is the degree of willingness to believe that the expectations will be met during online transactions ( Odusanya, Aluko, & Lal, 2020) without raising any risks ( Ryu & Ko, 2020).

In general, trust is crucial for FinTech users more than transactions on e-commerce or e-banking because of FinTech transaction uncertainty. Trust in the PTP lending platform is fundamental for capturing financial institutions' behavior and indicates that financial institutions welcome taking risks despite transaction uncertainty and believe that the lending platform will apply investment trading rules ( Yuwei, Zhihan, & Bin, 2017).

Ooi and Tan (2016) introduced the mobile technology acceptance model (MTAM), adding Technology Acceptance Model to the inadequacies in popular information technology models, which are adapted from electronic commerce literature ( Sharmin et al., 2021). In this model, MTAM MTAM is integrated with an extended valence framework to examine the factors impacting Malaysia's behavior intention Malaysia. It consists of mobile usefulness MU and ease of use mobile ease of use MEU, to determine smartphone credit card adoption. Both constructs are similar to perceived usefulness and perceived ease of use PEOU in the technology acceptance model (TAM) ( Sharmin et al., 2021). In addition, it proposed four more constructs to enhance overall predictability, mobile perceived security risk MPSR, mobile perceived compatibility mobile perceived compatibility MPC, mobile perceived trust (MPT MPT ), and mobile perceived financial resource MPFR. This framework represents a technology viewpoint ( Sharmin et al., 2021). The study surveyed 459 mobile users and tested using PLS-SEM PLS-SEM-ANN PLS-SEM-ANN, including linear and non-linear relationships.

Mobile technologies have become the mainstay of services delivery and business models. The MTAM model ( Ooi & Tan, 2016) defines the most crucial factors influencing intention when formulating a business model and delivering it through mobile technology. FinTech innovation uses mobile technology to give easy and smooth access to consumers and satisfy their needs. FinTech platforms started with web-based applications and recently introduced mobile technology-based to enable borrowers and lenders to exchange credit services. The lack of trust in the digital financing delivered through mobile platforms negatively affects the financial inclusion that drives digital finance, such as financial technology, in emerging and developing countries ( Ozili, 2018). However, the MPT variable was measured on the behaviour intention as a unidimensional factor in the MTAM model ( Ooi & Tan, 2016). Its effects on other factors such as mobile perceived ease of use, usefulness, economic benefit and convenience benefit were not identified. Consequently, there is a need to understand the other factors that affect the role of MPT and its influences on adopting the FinTech platform that uses mobile technology.

Chen et al. (2015) confirmed that perceived trust is the most significant construct of willingness to lend. In addition, they found that perceived risks ( Tan & Leby, 2016) had a negative impact on perceived trust. In many studies, such as mobile banking, perceived trust is a crucial factor predicting perception and intention toward adopting a behavior ( Alalwan, Dwivedi, & Rana, 2017). Additionally, perceived trust is considered a mediator that influences the positive benefits and intention ( Tang, Zhang, & Akram, 2019). Thus, this study defined MPT as the financial institution willing to rely on a FinTech company's mobile platforms to evaluate the borrowers and receive loan recommendations to select, with minimal risks by identifying the uncertainty. That platform will meet their credit expectations.

According to previous studies, perceived trust positively affects behavioral intention in various digital contexts such as e-commerce, internet banking, mobile banking, and mobile payments ( Ryu & Ko, 2020). In addition, perceived trust is only relevant in uncertain situations. Simultaneously it will reduce the uncertainty in a situation, i.e., trust happens when a party believes in another party can take actions that will result in a positive outcome for their interests and will not take action, which might result in an adverse effect ( Anderson & Narus, 1990). Furthermore, Ooi and Tan (2016) found that MPT increases intention for online payments, and it is the most influential construct on behavior intention. Moreover, trust increases the intention to use ( Mendoza-Tello, Mora, Pujol-López, & Lytras, 2019).

Odusanya et al. (2020) posit that intention is an outcome of trust. Therefore, perceived trust is an antecedent of intention. Trust is a critical indicator in human interactions, which enhances the relationships between the users and platforms, and it is a predominant factor in human behavior, influencing the intention ( Agag & El-Masry, 2016; Mendoza-Tello, Mora, Pujol-López, & Lytras, 2019). Furthermore, uncertainty is reduced by building high levels of Trust ( Ryu & Ko, 2020). Similarly, a lack of trust can negatively impact financial institutions using FinTech platforms using mobile technology ( Odusanya, Aluko, & Lal, 2020). Although many studies have explored the influences of trust on various digital business models, little attention has been given to the theoretical and empirical validation in a FinTech platform context ( Ryu & Ko, 2020).

### Research question

*Does the mobile perceived trust have mediating effects on the intention and adoption of Fintech innovations using mobile technology?*

We identified the need for a literature review in FinTech adoption intention and mobile trust. Our objective was to provide researchers and subject matter experts with a structured classification view of what has been produced in the MPT related to intention to adopt FinTech.

## Methods

A systematic literature review is widely adopted and used for research in technology and information systems to determine the art of crafting a research topic and develop evidence-based knowledge and guidance for researchers and subject matter experts in the investigated area.

**Figure 1.**
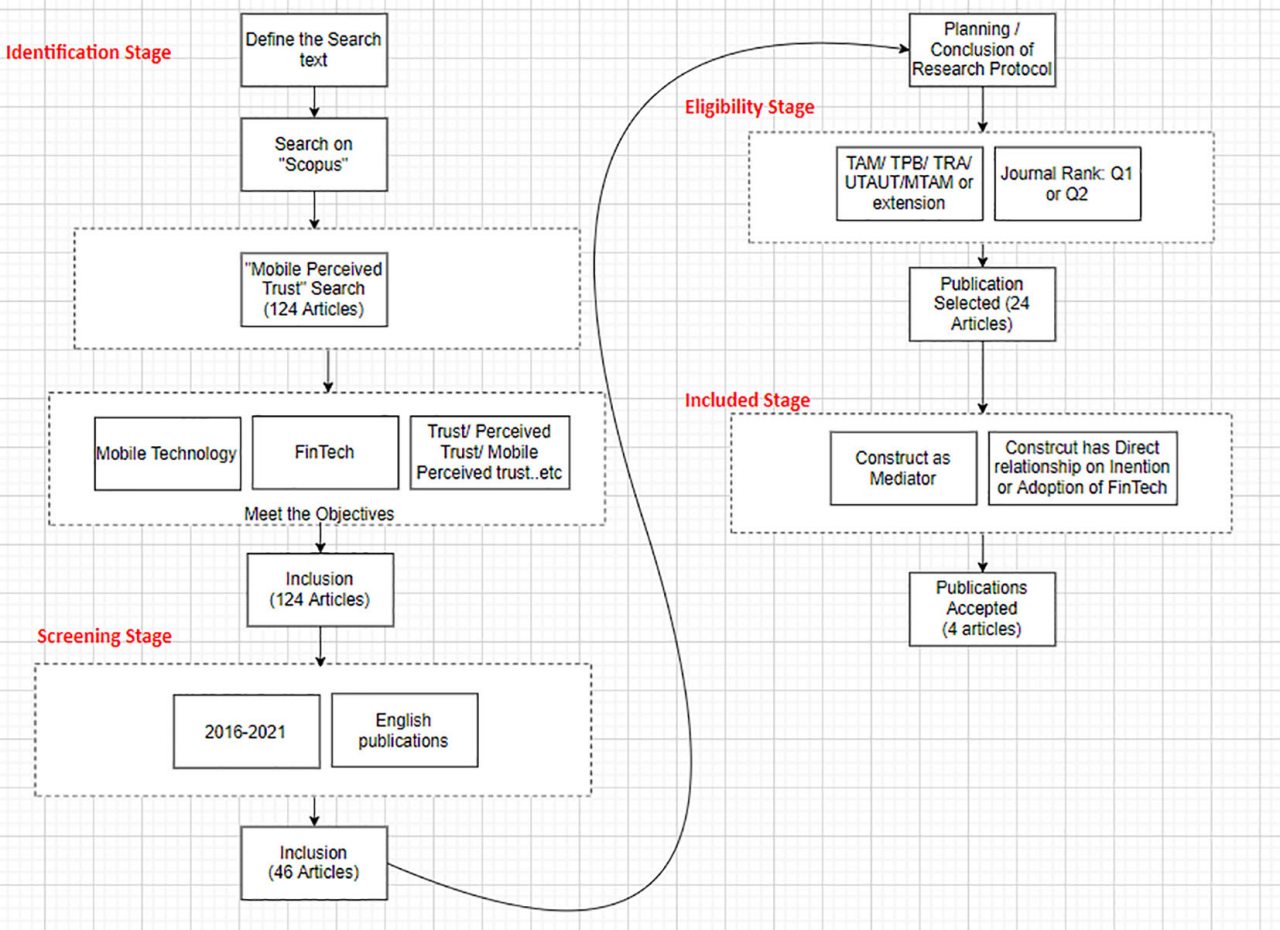
Search process.

Figure 1 shows the search process: identifying, screening, eligibility, and data extraction.

### Search strategy: identification stage

The Scopus database is used in this systematic literature review to ensure the quality of referenced articles. We formulated our research question by categorizing keywords according to population, outcomes, and context strategy. The research question was taken from the Fintech activities found in MTAM ( Ooi & Tan, 2016).

• Population: “Mobile Technology ” in the context of “FinTech”.
• Outcomes: 124 articles.

This research aims to find out articles that used “Mobile Perceived Trust” in contexts: “FinTech”, “Intention”, and “Adoption”. The keywords used for searching was defined as follows: “Mobile Perceived Trust ”, “Mobile Perceived Trust ” AND “FinTech”, “Mobile Perceived Trust ” AND “Intention”, and “Mobile Perceived Trust ” AND “Adoption”. The initial search with the keyword “Mobile Perceived Trust” yielded 124 articles from the Scopus database. Therefore, out of 124 articles, only 46 articles were found in these contexts for the keyword “Mobile Perceived Trust”.

### Selection criteria: screening stage

The research was narrowed to publications spanning from 2016 to 2021. The focus was on this period to ensure the quality of articles and to observe the FinTech trends clearly and accurately. This period also ensured a tremendous development of mobile phone technologies and applications in various fields. Furthermore, the digital generation (Z-generation) has reached the age of 16-19 years (born 1997-2012), in which they can own bank accounts and mature enough to use financial transactions using mobile technology. Publications of the type “Journal article” and “Conference papers” were selected, and those published in English were selected because they have undergone a rigorous peer-review process before publication ( Capobianco-Uriarte et al., 2019).

### Quality assessment: eligibility stage

The study is based only on original research articles and conference papers. All duplications were removed. The abstracts of the papers were revised to ensure the quality and relevance of the academic literature. The following exclusion criterion was to limit the papers to the following theories and their extensions: TAM ( Davis, 1989), theory of planned behaviour ( Ajzen, 1991), theory of reasoned action (Ajzen and Fishbein, 1980), Unified Theory of Acceptance and Use of Technology ( Venkatesh et al., 2003), MTAM ( Ooi & Tan, 2016). Furthermore, only articles published in journals ranked Q1 and Q2 were selected. After the filtration and quality assessment, 24 articles were eligible out of 124 papers.

### Data extraction

In this stage, four articles were selected ( Table 1). The criteria for selection were: 1) the construct must be a mediator (mobile perceived trust or trust or mobile trust). This coincided with the study aim and question; 2) the construct (MPT or trust or mobile trust) directly relates to the intention of behaviour adoption. Many researchers used MPT as an independent variable and directly measured its effect on the intention and adoption of behaviour. This research aims to select the articles that use MPT or mobile trust or trust as a mediator, mediating the effects of different variables such as benefits or risk variables on the intention or adoption of behaviour.

**Table 1.** Selected articles matching eligibility criteria.

Articles authors	Mediator	Mediating variables (independent variables)	Dependent variable
( To & Trinh, 2021)	Trust	Enjoyment	Behaviour intention
( Nguyen & Nguyen, 2020)	Trust	CSR variables (Economic responsibility, social responsibility, environmental responsibility) and perceived risk	Intention to use m-banking
( Vejačka & Štofa, 2017)	Trust	Perceived Security	Behaviour intention
( Gbongli, Xu, Amedjonekou, & Kovács, 2020)	Trust	Dispositional trust, Technology Trust, Vendor Trust	Adoption of mobile Financial Services

## Results

### Variables identified in the selected articles

The following are the variables mentioned in the selected articles, their effects and results obtained by the researchers.

*Mobile perceived trust*

Consumers perceived the trust in performing a financial transaction using mobile technology as worry-free and secure. They expect that the transactions using mobile technology will not be hacked and their information will be stored safely and secured. Due to a high degree of uncertainty, trust becomes a significant factor for users to use mobile technology ( Nguyen & Nguyen, 2020). Past studies found that trust significantly impacts consumer behaviour (intention and adoption) in uncertain environments ( To & Trinh, 2021). Furthermore, Gbongli et al. (2020) confirmed that customer’s trust positively impacts customer’s intention to use electronic banking.

*Perceived enjoyment*

**Figure 2.**
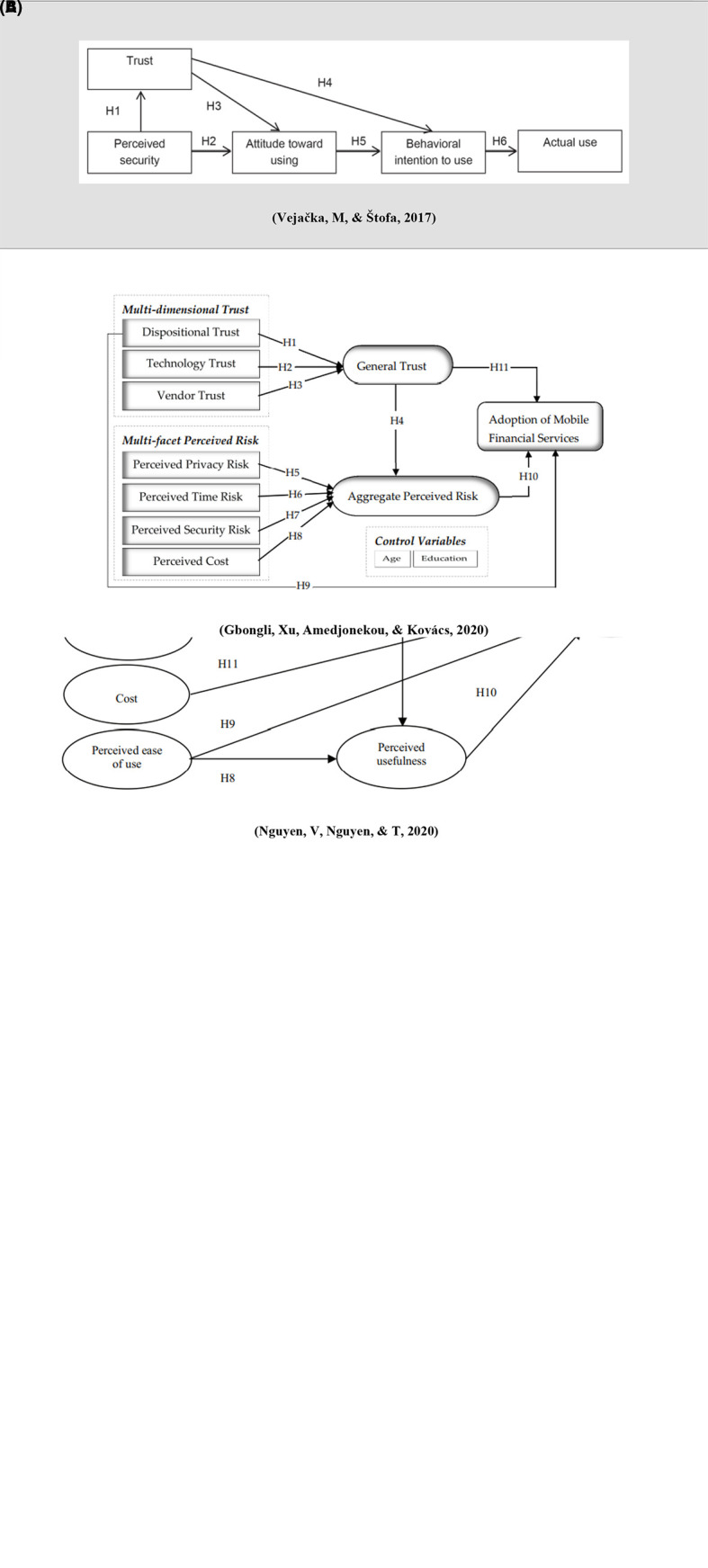
Research framework.

Venkatesh et al. (2012) defined perceived enjoyment as “the fun, pleasure, entertainment, or playfulness derived from using a technology”, and it found that it has a significant effect on consumer's technology acceptance. To & Trinh (2021) introduced perceived enjoyment as “the degree to which a person feels enjoyable when using e-wallets”. Previous studies empirically incorporated perceived enjoyment to the TAM and confirmed that this construct positively impacts behavioural intention (To). Furthermore, To & Trinh (2021) stated that an increase in perceived enjoyment decreases the worrying and improves customers trust in using technologies. To & Trinh (2021) found that the perceived enjoyment variable is an antecedent to consumer trust (β = 0.534, P-value <0.01), and this is consistent with Rouibah et al. (2016) ( Table 1 & Figure 2).

*CSR variables*

Corporate social responsibility (CSR) activities can affect customers’ trust and reduce scepticism. According to Karim et al. (2019), CSR is an emerging management model for an organization, and it contains a set of relationships, including owners, managers, and stakeholders interested in the evolution of that organization. Past studies reported that CSR impact positively consumer trust and lead to long-term affiliation. Moreover, a firm's ethical and legal responsibilities can positively affect consumers' trust ( Nguyen, V, Nguyen, & T, 2020). Furthermore, Karim et al. (2019) added that developing countries perceive more CRS challenges than developed countries.

*Perceived risk*

Perceived risk is one of the main obstacles and barriers affecting consumers' intention and adoption in using financial transactions through mobile technology ( Nguyen & Nguyen, 2020). It affects users' trust negatively in FinTech innovations. Past studies found perceived risk as the main barrier affecting the user’s intention and adoption of technology in Brazil, Iran, South Korea, Germany, Vietnam, and China ( Nguyen & Nguyen, 2020) ( Table 1 & Figure 2).

*Perceived security*

Perceived security is about cyber-security, cyber-threats and hacking of financial and personal information. Preventing security threats by enhancing the electronic security and safety of use will improve users' trust, increasing their intention and adoption to use financial services through mobile technology. Past studies found that perceived trust positively influences the user’s trust ( Vejačka & Štofa, 2017). Vejačka & Štofa (2017) found that perceived security has positive impact on customer's trust in electronic banking (β = 0.793, t = 11.224, p < 0.01) ( Table 1 & Figure 2).

*Dispositional trust*

Dispositional trust “explains the reason why some of us have a tendency to either trust or mistrust and doubt others” ( Gbongli, Xu, Amedjonekou, & Kovács, 2020). Therefore, it is essential for establishing initial trust. It found that it significantly influences users’ general trust in using mobile financial services (β = 0.207, p < 0.001) ( Gbongli, Xu, Amedjonekou, & Kovács, 2020) ( Table 1 & Figure 2).

*Technology trust*

Technology trust implies the relationship between the trust in using technology and the users. According to ( Gbongli, Xu, Amedjonekou, & Kovács, 2020). It is an antecedent of trust. It found that it has a strong positive impact on trust (β = 0.222, p < 0.001) ( Gbongli, Xu, Amedjonekou, & Kovács, 2020) ( Table 1 & Figure 2).

*Vendor trust*

Vendor trust implies the extent to which the consumers believe that the vendor will complete the transactional requirements in risky conditions ( Gbongli, Xu, Amedjonekou, & Kovács, 2020). Vendors' features such as integrity and ability are crucial trust features. Gbongli et al. (2020) found that it has a positive influence on general trust (β = 0.251, p < 0.001) ( Table 1 & Figure 2).

### Thematic analysis of the articles selected

This section discusses the results of the literature analysis on mobile perceived trust in the FinTech context using mobile technology. It covers terminology, thematic analysis of the methodology and content analysis related to the periods, publications, citations, and other information as main characteristics of the selected articles ( Table 2).

**Table 2.** Characteristics of selected articles.

Type	Author(s)	Title	Journal	Publisher	Rank	Year	DOI
Journal Article	( To & Trinh, 2021)	Understanding behavioral intention to use mobile wallets in Vietnam: Extending the tam model with trust and enjoyment	Cogent Business Management	Cogent OA (UK)	Q2	2021	10.1080/23311975.2021.1891661
Journal Article	( Nguyen & Nguyen, 2020)	An Integrated Model of CSR Perception and TAM on Intention to Adopt Mobile Banking	Journal of Asian Finance, Economics and Business	Korea Distribution Science Association (KODISA)	Q2	2020	10.13106/jafeb.2020.vol7.no12.1073
Journal Article	( Vejačka & Štofa, 2017)	Influence of security and trust on electronic banking adoption in Slovakia.	E+M Ekonomie a Management	Technical University of Liberec (Czech Republic)	Q2	2017	10.15240/tul/001/2017-4-010
Journal Article	( Gbongli, Xu, Amedjonekou, & Kovács, 2020)	Evaluation and Classification of Mobile Financial Services Sustainability Using Structural Equation Modeling and Multiple Criteria Decision-Making Methods	Sustainability	MDPI AG (Switzerland)	Q1	2020	10.3390/su12041288

*Terminology*

FinTech innovations typically have a high degree of uncertainty; therefore, trust becomes essential for consumers to obtain confidence. When consumers perceive mobile technology as a trustworthy platform, their intentions to adopt it will increase ( Shao, Zhang, Li, & Guo, 2019). According to Shareef et al. (2018), trust has an essential role in electronic transactions than traditional behavior. Shareef et al. (2018) defined trust as “ the degree to which users have attitudinal confidence for reliability, credibility, safety, and integrity of ”, FinTech innovations, “ from the technical, organizational, and social standpoints”.

*Publications per year*

A total of four articles were included in the final analysis. One was published in 2021, two in 2020, and one in 2017.

*Publications per FinTech business model*

FinTech business models are of four types: payment & remittance, insurance, lending, and investment ( Ryu, 2018; Alqaryouti, Siyam, Alkashri, & Shaalan, 2020). The payment and remittance business model in FinTech includes the payments for services and products through mobile technologies and using banks' online payments. FinTech lending business models consist of electronic credit platforms: crowdfunding, peer to peer lending, invoice funding, etc. FinTech investment business models or Robo-investment models are electronic platforms that use artificial intelligence for wealth management. The FinTech insurance platforms include registration, renewal, and maintenance. All the selected articles discussed mobile wallets, mobile banking, electronic banking, and mobile financial services. No article was found in lending or investment, or insurance. The four selected articles discussed payment and remittance financial technologies.

*Publications per journal*

A total of three articles were published in journals with rank Q2, and one was published in journals with rank Q1 ( Table 3). The ranking of journals represents the quality of journals that accepted and published the selected articles.

**Table 3.** Journals names and ranking.

Journal	Rank	Total
E+M Ekonomie a Management	Q2	1
Journal of Asian Finance, Economics and Business	Q2	1
Sustainability	Q1	1
Cogent Business & Management	Q2	1
Total		4

*Articles per methodology*

It was found that all selected articles (4) used an empirical survey data methodology.

*Theories used per article*

It was found that all selected articles (4) used the theory of TAM ( Davis, 1989) as the base theory for their research.

*Citations per year*

It was found that all selected articles (four) have been cited 31 times by other researchers and studies ( Table 4).

**Table 4.** Citations per article.

Journal	Total citations
E+M Ekonomie a Management	21
Journal of Asian Finance, Economics and Business	3
Sustainability	5
Cogent Business & Management	2
Total	31

*FinTech trends in the selected articles*

Table 5 highlights the main FinTech trends found in the selected articles. These trends are mobile banking and the sustainability of mobile financial services. Three out of four articles discuss mobile banking while only one article discusses the sustainability of FinTech. Furthermore, the sustainability article explored the adoption of behaviour while the others explored the intention of the behaviour.

**Table 5.** FinTech trends.

Trends	Issues
Mobile Banking	1. Influence of security and trust on electronic banking adoption ( Vejačka & Štofa, 2017; Nguyen & Nguyen, 2020; To & Trinh, 2021)
Sustainability	1. Mobile financial services sustainability ( Gbongli, Xu, Amedjonekou, & Kovács, 2020)

### Meta-analysis of the selected articles

This study performed a meta-analysis to explore mobile perceived trust (MPT) issues. It explored the research challenges and trends of the topics. Table 6 describes the challenges and issues addressed in the selected articles.

**Table 6.** Challenges discussed in selected articles.

Challenges	Issues
Motivation to use mobile payment & wallet	1. Information placement trust in the service provider 2. Security and privacy trust concerns 3. Ease of use impact consumer trust on the behavior intention 4. Usefulness impact consumer trust on the behavior intention
Building and maintaining consumer’s trust	1. Customer to provide personal or financial information 2. Enhancing customer level of enjoyment
The effects of social responsibility and environmental responsibility (CSR) on trust	1. CSR has a vital role in creating customer trust 2. Building customer trust is a costly and time-consuming exercise 3. This type of trust is built on accumulative experience 4. Customer’s trust may affect loyalty 5. Trust is a crucial element for decision making 6. Trust is an important factor for customer to obtain confidence 7. The perception of risk is a significant factor affecting trust
Customers perceive benefits from using mobile banking	1. Trust in information and communication technology affects the adoption of mobile banking 2. Faster and more reliable acquisition of information and support transparency enhances customer’s trust in using mobile banking 3. Security failure effects customer’s trust in using mobile banking 4. Bank supervision impact customer’s trust in using mobile banking 5. Customer’s trust in their banks affect using mobile banking
The sustainable development of mobile financial services (MFS)	1. Perceived risk effects negatively customer’s trust 2. Dispositional trust, technology trust and vendor trust are affecting adoption of MFS.

## Discussion

According to Vejačka et al. (2017), trust is essential in adopting mobile technology and directly affects consumers' intention to use mobile banking services. At the same time, To et al. (2021) confirmed that the adoption of mobile banking innovations requires building and maintaining customer trust because customers provide personal and financial information. Therefore, many concerns and issues, such as security and privacy ( Ooi & Tan, 2016) affect users’ acceptance of technology. Trust in mobile technology mediates perceived risk on the attitude toward mobile banking and the intention to adopt mobile banking ( Vejačka, M, & Štofa, 2017). In addition, it mediates enjoyment of perceived usefulness and the intention to adopt a mobile wallet ( To, A, Trinh, & T, 2021). On the other hand, sustainable development is a significant challenge facing people, and trust mediates corporate social responsibility (CSR) on adopting mobile banking ( Nguyen, V, Nguyen, & T, 2020). Gbongli et al. (2020) studied trust (general trust) as a multi-dimensional mediator for three types of antecedents: disposition trust, technology trust and vendor trust, which mediates them on adopting mobile financial services.

There is no doubt that trust in mobile technology is an essential factor in the adoption and acceptance of technology, especially related to finance and to carry out financial transactions through the mobile phone. The trust in mobile technology results from several factors (antecedent) such as the benefits gained, community acceptance, experience, information security, laws and regulations related to financial transactions through advanced technology and smartphone applications. It is also affected and weakened by the various risks that may occur in the absence of the financial technology ecosystem. The future is heading for more applications in financial technology driven by the preferences of the new generation: Z-generation and digital transformation. In conclusion, the factor of confidence in the acceptance of financial technology must be examined for its effects on intention and adoption using various frameworks and models such as MTAM ( Ooi & Tan, 2016).

The research results indicate that mobile perceived trust or mobile trust or trust in technology is used as a mediator and has a mediation effect on the intention and adoption. Nevertheless, few studies were conducted to explore the mediation effects of mobile perceived trust on the intention and adoption in the FinTech context.

However, the selected articles have several limitations. First, they focused mainly on one type of FinTech business model: mobile payment and remittance, ignoring other business models such as lending, insurance, and investment. Second, new theories and models such as the mobile technology acceptance model ( Ooi & Tan, 2016) was not considered, and instead, modified TAM ( Davis, 1989) was used. Third, these studies were conducted in Vietnam, South Korea, and Slovakia, limiting geographical areas. Fourth, the trust is used as consumer trust in the innovation, considering security and privacy, in general, mobile banking and financial services, while ignoring the perceived benefits mediation by trust in mobile technology (mobile perceived trust) toward intention and adoption of FinTech business model.

This study used the PRISMA approach as a systematic literature review and was limited to the extracted data from the Scopus database. Therefore, future studies need to consider other databases such as Google scholar, Emerald, WOS and others. By extracting all data from multiple databases can lead to more understanding of the role of mobile perceived trust or mobile trust or trust in technology in the FinTech context.

Further studies are required focusing on the mediation effects caused by mobile perceived trust ( Ooi & Tan, 2016) on intention and adoption of FinTech innovation, using both perceived benefits and perceived risks. In addition, other business models, such as lending or Robo-investment, are required to examine the mobile perceived trust mediation on the intention and adoption of FinTech innovations, using new frameworks and models such as MTAM ( Ooi & Tan, 2016) and benefit-risk framework ( Ryu, 2018), in developing countries and emerging economies countries.

## Data availability

### Underlying data

All data underlying the results are available as part of the article and no additional source data are required.

### Reporting guidelines

Zenodo: PRISMA checklist for ‘Mobile perceived trust mediation on the intention and adoption of FinTech innovations using mobile technology: a systematic literature review’, https://doi.org/10.5281/zenodo.5722717.

Data are available under the terms of the Creative Commons Attribution 4.0 International license (CC-BY 4.0).

## Footnotes

^1^
Retrieved from Forging-withbleeds.pdf (assets.kpmg).
