# Supplementary material for: Mobile perceived trust mediation on the intention and adoption of FinTech innovations using mobile technology: A systematic literature review
Source: F1000Res. 2022 Mar 2;10:1252. Originally published 2021 Dec 7. [Version 2] doi: 10.12688/f1000research.74656.2 (PMC9021664; doi:10.12688/f1000research.74656.2)
Supplement: Peer review history

# Peer review: Reviewer response for version 2

**DOI:** 10.5256/f1000research.121585.r125966

**Published:** 2022-04-13

**Authors:** Muhammad Imran Qureshi

**Affiliations:** 1 Faculty of Technology Management and Technopreneurship, Universiti Teknikal Malaysia Melaka, Durian Tunggal, Malaysia

Corrections have been done and the manuscript can be accepted in its current form.

Are the rationale for, and objectives of, the Systematic Review clearly stated?

Yes

Is the statistical analysis and its interpretation appropriate?

Yes

Are sufficient details of the methods and analysis provided to allow replication by others?

Yes

Are the conclusions drawn adequately supported by the results presented in the review?

Yes

Reviewer Expertise:

Systematic Literature review

I confirm that I have read this submission and believe that I have an appropriate level of expertise to confirm that it is of an acceptable scientific standard.

# Peer review: Reviewer response for version 2

**DOI:** 10.5256/f1000research.121585.r125967

**Published:** 2022-03-03

**Authors:** Sitara Karim

**Affiliations:** 1 Department of Business Administration, Faculty of Management Sciences, ILMA University, Karachi, Pakistan

There are no further comments.

Are the rationale for, and objectives of, the Systematic Review clearly stated?

Yes

Is the statistical analysis and its interpretation appropriate?

Yes

Are sufficient details of the methods and analysis provided to allow replication by others?

Yes

Are the conclusions drawn adequately supported by the results presented in the review?

Yes

Reviewer Expertise:

FinTech, Cryptocurrencies, Digital Assets, Blockchain, Financial Markets

I confirm that I have read this submission and believe that I have an appropriate level of expertise to confirm that it is of an acceptable scientific standard.

# Response

**Published:** 2022-03-03

**Authors:** Hatim Dawood

**Affiliations:** UCSI University, Malaysia

Thank you Dr Sitara.

# Peer review: Reviewer response for version 1

**DOI:** 10.5256/f1000research.78431.r102412

**Published:** 2022-02-08

**Authors:** Muhammad Imran Qureshi

**Affiliations:** 1 Faculty of Technology Management and Technopreneurship, Universiti Teknikal Malaysia Melaka, Durian Tunggal, Malaysia

The article, 'Mobile perceived trust mediation on the intention and adoption of FinTech innovations using mobile technology: A systematic literature review', attempted to map the literature to provide evidence for Mobile perceived trust as a meditator. The article followed the PRISMA standards.

 However, I believe the following improvements can enhance the readability of the article:

1. Only 4 articles were selected to provide a systematic review. Authors need to provide justification of this number or maybe they can broaden their search using different keywords.
2. Each table in the paper needs to be elaborated. Tables and figures are not self-explanatory and need to be explained in the article.
3. Minor language corrections are required.

Are the rationale for, and objectives of, the Systematic Review clearly stated?

Yes

Is the statistical analysis and its interpretation appropriate?

Yes

Are sufficient details of the methods and analysis provided to allow replication by others?

Yes

Are the conclusions drawn adequately supported by the results presented in the review?

Yes

Reviewer Expertise:

Systematic Literature review

I confirm that I have read this submission and believe that I have an appropriate level of expertise to confirm that it is of an acceptable scientific standard, however I have significant reservations, as outlined above.

# Response

**Published:** 2022-02-17

**Authors:** Hatim Dawood

**Affiliations:** UCSI University, Malaysia

Dear D Mohammed

 Thank you for your feedback.

 The remarks are clear and beneficial. I will amend the script and send it accordingly.

1. Only 4 articles were selected to provide a systematic review. Authors need to provide justification of this number or maybe they can broaden their search using different keywords. 
2. Each table in the paper needs to be elaborated. Tables and figures are not self-explanatory and need to be explained in the article.
3. Minor language corrections are required.

# Peer review: Reviewer response for version 1

**DOI:** 10.5256/f1000research.78431.r102414

**Published:** 2022-01-24

**Authors:** Sitara Karim

**Affiliations:** 1 Department of Business Administration, Faculty of Management Sciences, ILMA University, Karachi, Pakistan

Today, mobile technology has become the main backbone of FinTech innovations and acts as a channel for providing FinTech services that overcome all geographical and temporal barriers, thereby enhancing financial inclusion and developing banking services to suit the preferences of the generation Z "digital native" and digital transformation process. In this sense, enhancing trust in mobile technology will be the focus of the adoption of these innovations by users. I believe the paper is well-written and clearly expresses its contribution, tools, analysis, and results. The only shortcoming is the use of Nguyen and Nguyen (2020) article repetitively. I suspect that the article collection method suffers biasness.

 Regarding CSR, I would recommend adding these studies to enrich the support of its mediating effect:

- https://pubmed.ncbi.nlm.nih.gov/34826080/
- https://www.emerald.com/insight/content/doi/10.1108/CG-08-2020-0320/full/html
- https://www.emerald.com/insight/content/doi/10.1108/EDI-07-2020-0213/full/html
- https://ejournal.usm.my/aamj/article/view/91
- https://journals.sagepub.com/doi/10.1177/0972150920917773
- https://www.emerald.com/insight/content/doi/10.1108/JABS-07-2019-0214/full/html

I hope to see the revised version with better theoretical support on CSR. 

 All the best!

Are the rationale for, and objectives of, the Systematic Review clearly stated?

Yes

Is the statistical analysis and its interpretation appropriate?

Yes

Are sufficient details of the methods and analysis provided to allow replication by others?

Yes

Are the conclusions drawn adequately supported by the results presented in the review?

Yes

Reviewer Expertise:

FinTech, Cryptocurrencies, Digital Assets, Blockchain, Financial Markets

I confirm that I have read this submission and believe that I have an appropriate level of expertise to confirm that it is of an acceptable scientific standard, however I have significant reservations, as outlined above.

## References

[1] : Examining the role of gender diversity on ownership structure-sustainable performance nexus: fresh evidence from emerging markets. *Environ Sci Pollut Res Int* .2021; 10.1007/s11356-021-17459-6 10.1007/s11356-021-17459-6 34826080

[2] : Assessing the Governance Mechanisms, Corporate Social Responsibility and Performance: The Moderating Effect of Board Independence. *Global Business Review* .2020; 10.1177/0972150920917773 10.1177/0972150920917773

[3] : The Interaction Effect of Independent Boards on Corporate Governance-Corporate Social Responsibility (CG-CSR) and Performance Nexus. *Asian Academy of Management Journal* .2020;25(1) : 10.21315/aamj2020.25.1.4 10.21315/aamj2020.25.1.4

# Response

**Published:** 2022-02-17

**Authors:** Hatim Dawood

**Affiliations:** UCSI University, Malaysia

Dear Dr Sitara

 Thank you for your valued remarks.

 It is very generous of you to provide us with important references in this field(CSR). We will review these references and approve what is consistent with our research.

 As for this study (Nguyen V, Nquyen, and T), it is one of the four selected papers, using PRISMA process, and that was obtained and published in high-ranking journals (Q2), and accordingly, its repetition in the manuscript in order to clarify what was done in this research and the results it reached. Repetition was useful according to the context of the research.
